# Enhanced Photovoltaic Performance of Ternary Small Molecule/Polymer Bulk Heterojunction Solar Cells

**DOI:** 10.3390/mi17010097

**Published:** 2026-01-12

**Authors:** Soo Ah Nam, Jinwoo Lee, Joonwon Lim

**Affiliations:** 1 Department of Materials Science and Engineering, Korea Advanced Institute of Science & Technology (KAIST), Daejeon 34141, Republic of Korea; 2 Department of Information Display, College of Sciences, Kyung Hee University, Seoul 02447, Republic of Korea; 3 KHU-KIST Department of Converging Science and Technology, Kyung Hee University, Seoul 02447, Republic of Korea

**Keywords:** organic photovoltaics, ternary structure, small molecules, polymer donors

## Abstract

We report a notable enhancement in the performance of small-molecule-based organic photovoltaics (OPVs) through the use of a ternary blend comprising a small-molecule donor (DTS(FBTTh_2_)_2_), a polymer donor (PBDTTT-EFT), and a fullerene acceptor (PC_71_BM). By optimizing the composition of this ternary active layer, we achieved a significant increase in power conversion efficiency from 7.99% to 9.08%. This improvement is attributed to the broader light absorption spectrum and enhanced charge transport pathways provided by the polymeric donor. PBDTTT-EFT optimizes the nanomorphology and ordering of the bulk heterojunction films and forms a cascade energy level that enhances charge carrier mobility. Our results demonstrate that semiconducting polymer donors can effectively control light absorption, charge transport, and exciton dissociation by optimizing morphology and crystallinity. This approach offers new possibilities for advancing the performance of various optoelectronic devices through strategic use of different semiconducting polymer donors.

## 1. Introduction

The demand for a variety of wearable, lightweight, and flexible power supply devices is increasing, particularly for applications in body-attached devices, small robots, and real-time sensor systems. Organic photovoltaics (OPVs) are garnering significant attention as futuristic flexible energy supply devices due to their distinct advantages, including their lightweight nature, straightforward solution processing, and inherent compatibility with mechanically flexible device architectures [1,2,3,4]. Among the various types of OPVs, small molecule-based solar cells have attracted substantial research interest due to their balanced carrier mobility and solution processability with common organic solvents [5,6,7,8,9,10]. Despite these advantages, the efficiency of small molecule-based organic solar cells remains inferior to polymer-based organic solar cells [11,12,13]. Although small-molecule donors generally exhibit higher intrinsic crystallinity than polymer donors, solution-processed small-molecule bulk heterojunction films often suffer from sub-optimal nanoscale morphology arising from pronounced aggregation and limited miscibility with acceptor materials. Such morphology-related issues can lead to overly large crystalline domains, incomplete percolation pathways, and, consequently, insufficient charge-carrier mobility, which remain pivotal challenges for further enhancing device performance.

To date, numerous strategies have been investigated to enhance the performance of small molecule-based organic solar cells, including the incorporation of molecular or nanoscale additives into the small molecular active layers [14,15,16,17,18,19], the introduction of interfacial charge transport layers [20,21,22,23], the modification of device architecture [24,25,26,27,28,29,30], and the utilization of diverse acceptors [31,32]. Specifically, solvent additives such as 1,8-diiodooctane (DIO), chloronaphthalene, or polymeric additives like polydimethylsiloxane [17] have been shown to alter the morphological length scales of the donor and acceptor phases and crystalline structures of the small molecule-based active layers. These changes lead to enlarged interfaces between donors and acceptors as well as enhanced charge carrier mobility within the organic bulk heterojunction (BHJ) layer. The optimal morphologies of the active layers facilitate efficient exciton dissociation and rapid charge carrier transport, resulting in high power conversion efficiency (PCE) of the OPV. Additionally, incorporating a polymer donor component into a ternary small-molecule/polymer blend, which generally exhibits higher charge-carrier mobility than small-molecule donors, can improve the charge-transport properties of small-molecule-based active layers [14,16,33]. Furthermore, the introduction of semiconducting polymers species, which can absorb at longer wavelengths, can contribute to the enhancement in PCE by extending the range of light absorption of the small molecule-based organic solar cells [29,34,35].

In this study, we demonstrate a remarkable enhancement in the performance of small molecule-based organic solar cells by introducing a ternary blended active layer composed of semiconducting polymer species. The incorporation of poly[4,8-bis(5-(2-ethylhexyl)thiophen-2-yl)benzo[1,2-b;4,5-b′]dithiophene-2,6-diyl-alt-(4-(2-ethylhexyl)-3-fluorothieno[3,4-b]thiophene-)-2-carboxylate-2-6-diyl)] (PBDTTT-EFT) into 7,7′-(4,4-bis(2-ethylhexyl)-4H-silolo[3,2-b:4,5-b′]dithiophene-2,6-diyl)bis(6-fluoro-4-(5′-hexyl-[2,2′-bithiophen]-5-yl)benzo[c][1,2,5]thiadiazole) (DTS(FBTTh_2_)_2_) BHJ films with [6]-phenyl C_71_ butyric acid methyl ester (PC_71_BM) acceptor was strategically designed, and the resulting performance of the OPVs was measured and analyzed. The PBDTTT-EFT semiconducting polymer facilitated enhanced charge transport of the ternary blended active layer by achieving optimal nanomorphology and improved ordering within BHJ films. Moreover, PBDTTT-EFT contributed to forming a cascade energy level, which allowed for the broadening of the light absorption spectrum as well as enhancing the charge carrier mobility. The ternary blended BHJ organic solar cell achieved the maximum power conversion efficiency (PCE) of 9.08%.

## 2. Materials and Methods

### 2.1. Materials

7,7′-[4,4-Bis(2-ethylhexyl)-4*H*-silolo[3,2-*b*:4,5-*b*′]dithiophene-2,6-diyl]bis[6-fluoro-4-(5′-hexyl-[2,2′-bithiophen]-5-yl)benzo[*c*][1,2,5]thiadiazole] (DTS(FBTTh_2_)_2_, 99%), Poly({4,8-bis[(4-(2-ethylhexyl)thiophen-2-yl)]benzo[1,2-b:4,5-b′]dithiophene-2,6-diyl}{3-fluoro-2-[(2-ethylhexyl)carbonyl]thieno[3,4-b]thiophenediyl}) (PBDTTT-EFT, 99%), [6]-Phenyl C_71_ butyric acid methyl ester (PC_71_BM, 99%) were purchased from 1-Materials (Dorval, QC, Canada). Chlorobenzene (C_6_H_5_Cl, 99.8%), 1,8-Diiodooctane (I(CH)_8_I, 98%), Calcium (Ca, 99%), Aluminum (Al, 99.999%) were purchased from Sigma-Aldrich (St. Louis, MO, USA). PEDOT:PSS (Clevios P VP AI 4083) was used for experiments.

### 2.2. Fabrication of Organic Solar Cells

Commercial ITO-coated glass substrates (sheet resistance of 10–15 Ω/sq, ITO thickness ≈ 150 nm, Samsung Corning, Corning, NY, USA) were used as transparent anodes. The ITO glass substrates were cleaned by sonication in deionized (DI) water, acetone and 2-propanol. The cleaned ITO substrates were then exposed to UV-ozone for 20 min to create a hydrophilic surface. Subsequently, a PEDOT:PSS solution was spin-coated onto the ITO substrates at 3500 rpm. A DTS(FBTTh_2_)_2_:PBDTTT-EFT:PC_71_BM solution was prepared by dissolving DTS(FBTTh_2_)_2_ and PC_71_BM in a 3:2 mass ratio using chlorobenzene as the solvent, with the addition of 0.4 vol% DIO, to achieve a concentration of 35 mg/mL. For the preparation of the ternary solution, the ratio of PBDTTT-EFT was varied to 0.1, 0.2, 0.3, 0.4, and 0.5. Both the binary and ternary solutions were thoroughly dissolved at 60 °C and 550 rpm within a glove box. Prior to spin-coating, the solutions were annealed at 90 °C for 15 min. The solutions were spin-coated onto substrates pre-coated with PEDOT:PSS at 2000 rpm within the glove box, followed by immediate post-annealing at 80 °C for 10 min. After deposition and thermal annealing of the active layer, the counter-electrode was formed by thermal evaporation of calcium (Ca) and aluminum (Al) in sequence. Ca with a thickness of 20 nm was first deposited onto the active layer, followed by an Al overlayer with a thickness of 80 nm, under a base pressure below 3 × 10^−6^. Torr using a shadow mask to define the device area. The resulting device structure was therefore ITO/PEDOT:PSS/DTS(FBTTh_2_)_2_:PBDTTT-EFT:PC_71_BM/Ca/Al, with an active area of 0.04 cm^2^ used for all photovoltaic measurements.

### 2.3. Characterizations

The organic solar cells were characterized for current–voltage measurements using a solar simulator (ABET, LS-150-Xe, Baltimore, MD, USA), a source meter (Keithley, 2400, Solon, OH, USA), and a radiometer photodetector (International Light Tech., Inc., ILT1400-A, Peabody, MA, USA). The external quantum efficiency (EQE) and internal quantum efficiency (IQE) were measured using an IPCE measurement system (Newport IQE200 system, Oriel Instrument, Irvine, CA, USA). Absorption spectra were obtained using a UV-vis absorption spectrometer (UV-3600, Shimadzu, Kyoto, Japan). PL and TRPL measurements were performed with time-correlated single-photon counting (TCSPC) system (iHR320, Horiba Jobin Yvon Inc., Edison, NJ, USA). Two-dimensional GIWAXS pattern were obtained from X-ray synchrotron (3C beam-line, Pohang Accelerator Lab, Pohang, Republic of Korea). To estimate the mobility of electrons and holes, the space charge limited current (SCLC) model was employed.

## 3. Results and Discussion

### 3.1. Ternary Blend Solar Cells

**Figure 1:**
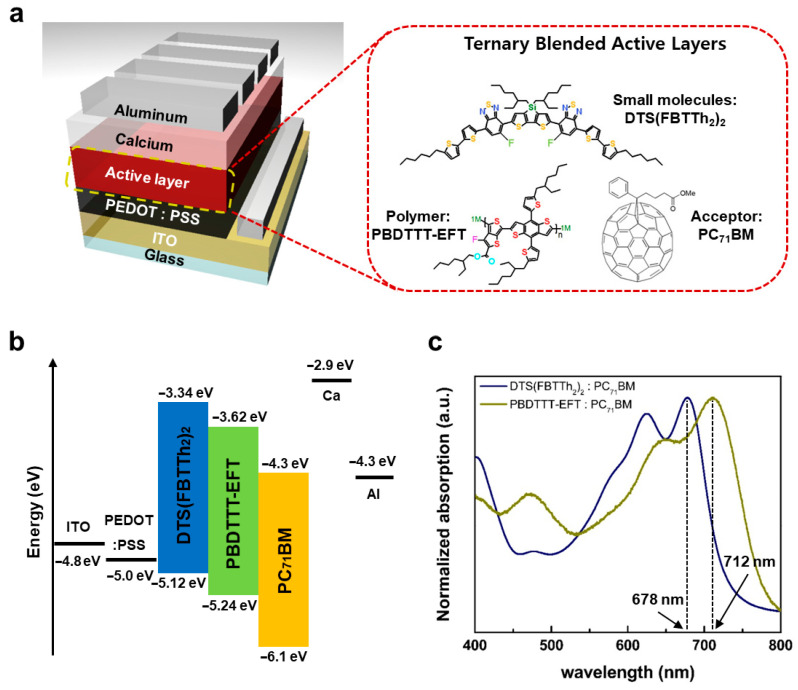
Characterization of materials in ternary blend solar cells. (**a**) Device structure (left) and chemical structures of materials (right) in ternary blend solar cells: DTS(FBTTh_2_)_2_, PBDTTT-EFT and PC_71_BM. (**b**) Energy-level diagram of ternary blend solar cells. (**c**) Normalized UV-VIS absorption spectra of DTS(FBTTh_2_)_2_:PC_71_BM film and PBDTTT-EFT:PC_71_BM film.

Figure 1a illustrates the structure of a ternary blended solar cell with an active layer composed of the small molecules DTS(FBTTh_2_)_2_, the semiconducting polymer PBDTTT-EFT, and the PC_71_BM acceptor. The electronic properties of these materials, characterized by their HOMO and LUMO levels, are crucial for understanding electron flow within the device. Specifically, the HOMO and LUMO levels of DTS(FBTTh_2_)_2_ are −5.12 eV and −3.34 eV, respectively [36]. In comparison, PBDTTT-EFT exhibits levels of −5.24 eV and −3.62 eV [37], while PC_71_BM shows levels at −6.1 eV and −4.3 eV [21,22]. The introduction of PBDTTT-EFT into the small-molecule active layer composed of DTS(FBTTh_2_)_2_ and PC_71_BM induces a cascade energy level diagram, as detailed in Figure 1b, which facilitates efficient charge transfer. Figure 1c shows UV-VIS absorption spectrum of DTS(FBTTh_2_)_2_:PC_71_BM and PBDTTT-EFT:PC_71_BM thin films. DTS(FBTTh_2_)_2_:PC_71_BM films exhibit two peaks at 624 nm and 678 nm, originating from the vibronic structure of DTS(FBTTh_2_)_2_:PC_71_BM film. Notably, the maximum absorption peak for PBDTTT-EFT:PC_71_BM film is observed at 712 nm. The red-shift in the absorption spectra of PBDTTT-EFT:PC_71_BM is due to the small HOMO-LUMO gap of PBDTTT-EFT compared to that of DTS(FBTTh_2_)_2_. This indicates that the incorporation of PBDTTT-EFT into the typical DTS(FBTTh_2_)_2_:PC_71_BM active layer can enhance PCE by broadening the absorption range in the solar spectrum.

### 3.2. Optical Characteristics of Ternary Blend Solar Cells

**Figure 2:**
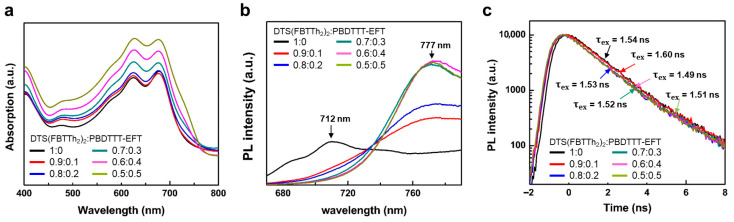
Optical characteristics of ternary blend solar cells. (**a**) UV-VIS absorption spectra of DTS(FBTTh_2_)_2_:PBDTTT-EFT:PC_71_BM films. (**b**) Steady-state photoluminescence spectra of DTS(FBTTh_2_)_2_:PBDTTT-EFT films. (**c**) Time-resolved photoluminescence profile of DTS(FBTTh_2_)_2_:PBDTTT-EFT solutions.

Figure 2a presents UV-VIS absorption spectra of all DTS(FBTTh_2_)_2_:PBDTTT-EFT:PC_71_BM films with different DTS(FBTTh_2_)_2_:PBDTTT-EFT weight ratios. The ratio of PBDTTT-EFT was precisely controlled from 0.1 to 0.5 relative to DTS(FBTTh_2_)_2_. As the ratio of PBDTTT-EFT increases, the light absorption of the active layers is continuously enhanced across the entire wavelength range from 400 nm to 800 nm. The noticeable increase in light absorption above 700 nm is attributed to the addition of PBDTTT-EFT, which has a maximum absorption peak at 712 nm. The change in the light absorption spectrum indicates that the ternary blended layer of DTS(FBTTh_2_)_2_:PBDTTT-EFT:PC_71_BM is definitely advantageous for broadening light absorption efficiency with comparison to DTS(FBTTh_2_)_2_:PC_71_BM. Figure 2b shows the steady-state photoluminescence (PL) spectrum of DTS(FBTTh_2_)_2_:PBDTTT-EFT films with varying weight ratios of PBDTTT-EFT on PEDOT:PSS/ITO substrates. Interestingly, the incorporation of a small amount (0.1 ratio) of PBDTTT-EFT with DTS(FBTTh_2_)_2_ causes a drastic red-shift in the maximum peak position of DTS(FBTTh_2_)_2_ from to 712 nm to 777 nm in PL spectrum. The intensity of the peak at 777 nm gradually increases with the PBDTTT-EFT ratio, implying that energy transfer between DTS(FBTTh_2_)_2_ and PBDTTT-EFT is favorable [28]. Figure 2c present the time-resolved PL profile of DTS(FBTTh_2_)_2_:PBDTTT-EFT solutions. A 405 nm wavelength excitation source (λ_ex_) was used and the emission (λ_em_) at 777 nm was detected. The exciton lifetime (τ_ex_) of pure DTS(FBTTh_2_)_2_ solution (0.025 mg mL^−1^) was measured to be 1.54 ns. As the ratio of PBDTTT-EFT to DTS(FBTTh_2_)_2_ increased from 0.1 to 0.5, τ_ex_ changed to 1.60, 1.53, 1.52, 1.49, and 1.51 ns, respectively. The variation in τ_ex_ for each case is not apparent and dependent on the ratio of PBDTTT-EFT to DTS(FBTTh_2_)_2_, indicating that the introduction of PBDTTT-EFT to DTS(FBTTh_2_)_2_:PC_71_BM has a negligible effect on the exciton dissociation in the ternary blended films.

### 3.3. Current–Voltage (I-V) Characteristics of Ternary Blend Solar Cells

**Figure 3:**
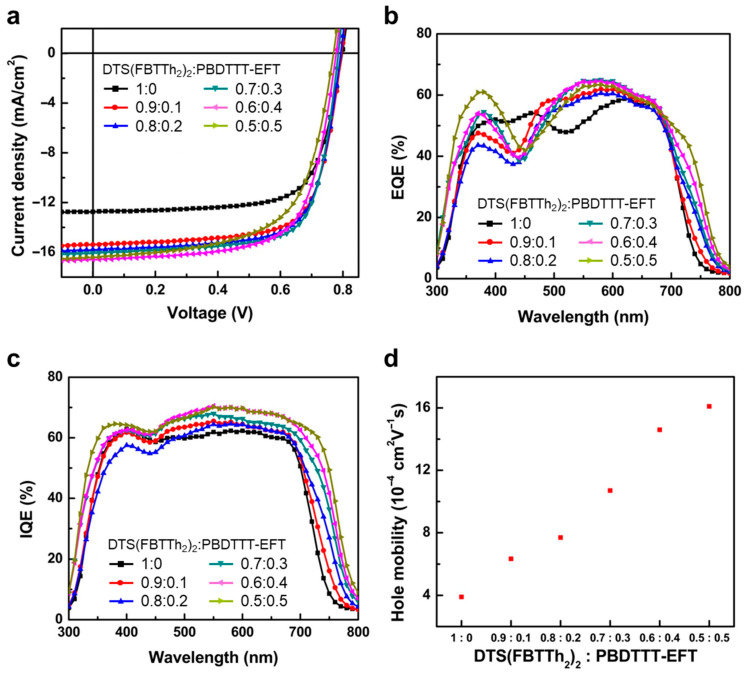
Current–voltage (I-V) characteristics of ternary blend solar cells. (**a**) J-V characteristics of the highest ternary blend solar cells. (**b**) EQE and (**c**) IQE of ternary blend solar cells. (**d**) Hole mobility of hole-only devices calculated with SCLC models.

The photovoltaic performance of the ternary system was investigated using simple device structure: indium tin oxide (ITO)/poly(3,4-ethylenedioxythiophene):poly(styrenesulphonate) (PEDOT:PSS)/DTS(FBTTh_2_)_2_:PBDTTT-EFT:PC_71_BM/Ca/Al. The corresponding photovoltaic performance parameters are summarized in Table 1. Figure 3a illustrates the representative current density versus voltage (*J*–*V*) characteristics of devices with different DTS(FBTTh_2_)_2_:PBDTTT-EFT weight ratios (0, 0.1, 0.2, 0.3, 0.4, and 0.5 of PBDTTT-EFT incorporation) under simulated AM 1.5 G illumination at 100 mW cm^−2^. Photovoltaic devices with different PBDTTT-EFT concentrations showed a consistent thickness around 100 nm. The DTS(FBTTh_2_)_2_:PC_71_BM control device started with a J_sc_ of 12.72 mA cm^−2^, an open circuit voltage (V_oc_) of 0.80 V, a fill factor (FF) of 70.86%, and a PCE of 7.99%. By adding a 0.1 ratio of PBDTTT-EFT to the host binary blend, the PCE was enhanced to 8.15% with a J_sc_ of 15.38 mA cm^−2^, a V_oc_ at 0.80 V, and an FF at 70.85%. Devices with a 0.3 ratio of PBDTTT-EFT incorporation showed the best solar cell performance, with a J_sc_ of 16.06 mA cm^−2^, a V_oc_ of 0.78 V, and an FF of 72.08%, resulting in a promising PCE of 9.08%. This is more than 13% enhancement in PCE compared with the reference device. An average PCE of 8.70 ± 0.16% was achieved over 10 identical devices under this condition. However, the photovoltaic devices with more than 0.3 ratio of PBDTTT-EFT gradually decline in performance, showing a PCE of 8.77% and 7.91%, with a 0.4 and 0.5 ratio of PBDTTT-EFT, respectively. As the weight ratio of PBDTTT-EFT increased from 0.1 to 0.5, the V_oc_ decreased from 0.797 V to 0.769 V. Yjos is associated with the combined effects of composition-dependent changes in recombination and device resistances in the ternary architecture. Meanwhile, the J_sc_ notably improved from 12.72 mA/cm^2^ for DTS(FBTTh_2_)_2_:PC_71_BM control device to a maximum J_sc_ of 16.57 mA/cm^2^ with the addition of 40% PBDTTT-EFT. The significant improvement in PCE with PBDTTT-EFT is primarily attributed to the enhancement of J_sc_ with higher external quantum efficiency (EQE) value in the 500–750 nm range, as shown in Figure 3b. The maximum EQE of the reference device of DTS(FBTTh_2_)_2_:PC_71_BM was 59.1%, whereas DTS(FBTTh_2_)_2_:PBDTTT-EFT(40%):PC_71_BM devices demonstrated 66.2%. Overall integration of the EQE spectrum yields J_sc_ values of 12.4 mA cm^−2^ for the reference device and 16.1 mA cm^−2^ for the PBDTTT-EFT(0.4 ratio) device. These values are consistent with the J_sc_ values in J–V measurements within a 3% error margin. This improvement in EQE aligns well with the tendency of UV-VIS absorption. As shown in Figure 3c, the internal quantum efficiency (IQE) values of OPVs were enhanced across the overall wavelength with PBDTTT-EFT, indicating that charge collection, including the dissociation of photo-generated excitons and subsequent charge transport, is efficiently achieved in the devices [35]. This confirms that the enhanced light absorption and improved charge transport in the presence of PBDTTT-EFT significantly contribute to the overall charge collection enhancement.

The enhancement of charge mobility by PBDTTT-EFT was confirmed through hole mobility measurements in hole-only devices using the space charge limited current (SCLC) model (Supplementary Figure S1 and Supplementary Table S2). Hole-only devices were composed of ITO, PEDOT:PSS, active layer, WO_3_, and Al layers. As shown in Figure 3d, the hole mobility in devices with semiconducting polymer donor PBDTTT-EFT increased significantly from 3.90 × 10^−4^ cm^2^ V^−1^ s^−1^ to 1.61 × 10^−3^ cm^2^ V^−1^ s^−1^ as the ratio of PBDTTT-EFT increased. This enhancement of hole mobility within the ternary blended active layers is attributed to the intrinsic electrical properties of polymeric semiconducting donors and the morphological changes in ternary blended active layers, which induce proper BHJ formation [36,37]. In ternary blended systems, polymer species can enhance carrier transport due to their superior carrier mobility compared to small organic molecules [38]. Additionally, the incorporation of polymer donors facilitates BHJ, providing more pathways for dissociated charge carriers to move through the polymeric species toward the electrodes. The increase in hole mobility directly enhances charge transport, resulting in improvements in J_sc_ and, ultimately, PCE.

The evolution of the series and parallel resistances (R_s_A and R_p_A) with composition further clarifies the origin of the PCE trends summarized in Table 1. The series resistance R_s_A decreases from 6.20 Ω cm^−2^ for the binary DTS(FBTTh_2_)_2_:PC_71_BM device to a minimum of 4.55 Ω cm^−2^ at DTS(FBTTh_2_)_2_:PBDTTT-EFT = 0.7:0.3, and then increases again to 5.98 Ω cm^−2^ at 0.5:0.5. This reduction in R_s_A at an intermediate PBDTTT-EFT content is consistent with the enhanced hole mobility (Figure 3d) and improved percolation pathways in the ternary blends, and it correlates with the highest fill factor (72.08%) and PCE (9.08%) observed at the 0.7:0.3 composition. In contrast, the partial increase in R_s_A at 0.5:0.5 contributes to the significant FF decrease to 62.77%, which limits the PCE despite the relatively high J_sc_.

The parallel resistance (R_p_A) shows a monotonic decrease from 2153.2 Ω cm^−2^ in the binary reference to 908.3 Ω cm^−2^ at 0.7:0.3 and further down to 553.8 Ω cm^−2^ at 0.5:0.5. This behavior indicates that, as the PBDTTT-EFT fraction increases, additional leakage or recombination pathways are introduced. Up to a PBDTTT-EFT ratio of 0.3–0.4, the beneficial effects of broadened absorption, improved charge transport, and lower R_s_A dominate, resulting in higher J_sc_ and PCE. However, at 0.5:0.5, the strongly reduced R_p_A suggests enhanced shunt or recombination losses, which, combined with the increased R_s_A, leads to a substantial reduction in FF, and hence a lower PCE compared to the optimally balanced 0.7:0.3 composition. Overall, the resistance analysis confirms that the best device performance is achieved when the ternary blend provides both low series resistance for efficient charge extraction and sufficiently high parallel resistance to suppress leakage and recombination.

In addition, the photovoltaic parameters in Table 1 reveal how the ternary composition governs device performance. With increasing PBDTTT-EFT content, J_sc_ increases markedly from 12.72 to 16.06–16.57 mA cm^−2^, which correlates with the broadened and red-shifted absorption and the enhanced EQE/IQE in the 500–750 nm range (Figure 2 and Figure 3b,c). In contrast, V_oc_ decreases slightly from 0.797 to 0.769 V, consistent with the deeper HOMO level of PBDTTT-EFT and the resulting reduction in the effective donor–acceptor energy offset. The fill factor initially improves from 70.86% to 72.08% at DTS(FBTTh_2_)_2_:PBDTTT-EFT = 0.7:0.3, where the series resistance R_s_A reaches a minimum and the parallel resistance R_p_A remains sufficiently high, reflecting efficient charge extraction and limited leakage. At higher PBDTTT-EFT fractions (0.4–0.5), however, R_s_A increases and R_p_A drops to 553.8 Ω cm^−2^, leading to enhanced recombination and a pronounced FF loss despite the highest hole mobility. Consequently, the maximum PCE of 9.08% is obtained at the 0.7:0.3 composition, where the ternary blend achieves an optimal balance between increased J_sc_, only modest V_oc_ loss, and favorable resistance-limited charge transport.

### 3.4. Morphological Characteristics of Ternary Blend Films

**Figure 4:**
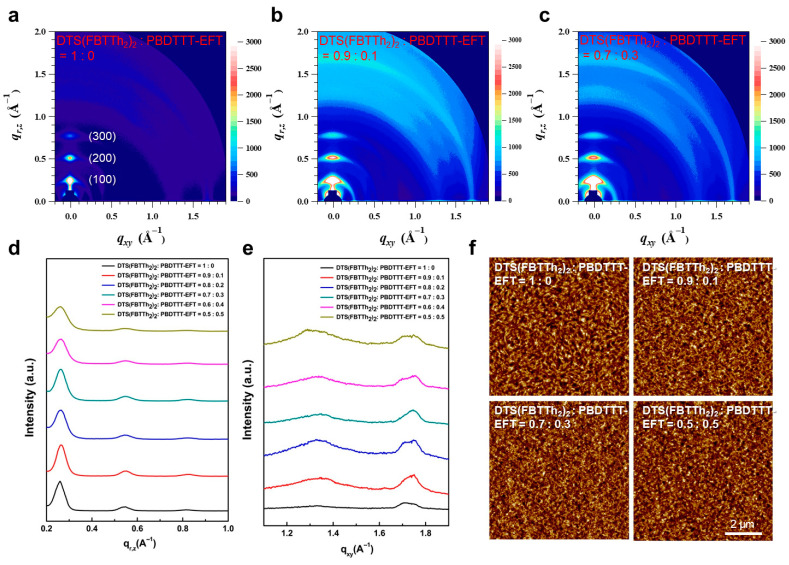
Morphological characteristics of ternary blend films: (**a**–**c**) 2D GIWAXS analysis of ternary blend solar cell films: (**a**) DTS(FBTTh2)2:PBDTTT-EFT = 1:0; (**b**) DTS(FBTTh2)2:PBDTTT-EFT = 0.9:0.1; (**c**) DTS(FBTTh2)2:PBDTTT-EFT = 0.7:0.3; (**d**) out-of-plane (q_r,z_) line cut of GIWAXS patterns; (**e**) in-plane (q_x,y_) line cut of GIWAXS patterns; (**f**) AFM images of ternary blend films.

Two-dimensional (2D) grazing incidence wide-angle X-ray scattering (GIWAXS) measurements were carried out on DTS(FBTTh_2_)_2_:PBDTTT-EFT: PC_71_BM films with different PBDTTT-EFT ratios to elucidate how the ternary composition modifies the molecular packing and orientation (Figure 4a–e and Figure S2). In the out-of-plane (q_r,z_) line cuts (Figure 4d), distinct (h00) reflections of DTS(FBTTh_2_)_2_ are clearly observed for all compositions. The positions of these peaks remain essentially unchanged upon introducing PBDTTT-EFT, indicating that the lamellar stacking distance of the small-molecule donor is preserved in the ternary blends. At the same time, the intensity of the (h00) reflections gradually decreases with increasing PBDTTT-EFT content, which suggests a reduction in large, highly oriented DTS(FBTTh_2_)_2_ crystallites and a transition towards smaller and more finely distributed crystalline domains.

In the in-plane (q_x,y_) profiles (Figure 4e), bimodal scattering peaks near q_x,y_ ≈ 1.70 and 1.76 Å^−1^ are assigned to π–π stacking of DTS(FBTTh_2_)_2_ and the crystallization of PC_71_BM, respectively [39,40]. Notably, the PC_71_BM-related peaks near 1.3 and 1.76 Å^−1^ are significantly enhanced by the incorporation of PBDTTT-EFT, indicating improved fullerene packing and the formation of more continuous electron-transport networks in the ternary active layer. Together with the known tendency of PBDTTT-EFT to adopt a predominantly face-on orientation on PEDOT:PSS substrates in optimized blends, these results suggest that the ternary donor phase maintains a substantial fraction of face-on (or mixed) orientational order in which lamellar stacking occurs mainly parallel to the substrate, while π–π stacking has a significant out-of-plane component that facilitates vertical charge transport to the electrodes.

AFM images (Figure 4f and Figure S4) corroborate this picture: the binary DTS(FBTTh_2_)_2_:PC_71_BM film exhibits randomly oriented fibrillar features, whereas these fibrils disappear and the domain morphology becomes more uniform upon the addition of PBDTTT-EFT. The crystallite size in the out-of-plane direction, estimated from the GIWAXS data using the Scherrer equation, decreases from approximately 10 nm in the binary blend to about 7 nm at higher PBDTTT-EFT content. Such domain sizes are comparable to typical exciton diffusion lengths and thus enable efficient exciton dissociation while maintaining percolated donor and acceptor pathways.

Collectively, the GIWAXS and AFM results demonstrate that PBDTTT-EFT modulates the BHJ morphology by reducing overly large DTS(FBTTh_2_)_2_ domains, enhancing PC_71_BM crystallinity, and promoting a face-on—like the packing of the donor phase. This optimized nanoscale morphology, in combination with the broadened absorption provided by PBDTTT-EFT, leads to improved charge generation and vertical charge transport, which is directly reflected in the enhanced J_sc_ and EQE/IQE values of the ternary devices (Table 1 and Figure 3b,c).

## 4. Conclusions

We demonstrated a significant enhancement in the performance of small molecule-based organic solar cells by introducing semiconducting polymer donors. The incorporation of PBDTTT-EFT into the DTS(FBTTh_2_)_2_:PC_71_BM active layer resulted in a remarkable improvement of PCE from 7.99% to 9.08%. This enhancement was primarily due to the broader light absorption range and improved charge transport pathways facilitated by the polymer donor. The use of PBDTTT-EFT not only optimized the nanomorphology and ordering within the BHJ films but also formed a cascade energy level that enhanced charge carrier mobility. Our findings suggest that the semiconducting polymer donor component in the ternary blend effectively controls light absorption, charge transport, and exciton dissociation by optimizing the active-layer morphology and crystallinity. This approach opens up new possibilities for enhancing the performance of various optoelectronic devices through the strategic use of different semiconducting polymer donors.

## Figures and Tables

**Table 1:** Device characteristics of organic solar cells with different ratio of DTS(FBTTh_2_)_2_:PBDTTT-EFT.

DTS(FBTTh_2_)_2_:PBDTTT-EFT	V_OC_	J_SC_	FF	R_S_A	R_P_A	PCE max.	PCE ave.
	[V]	[mA cm^2^]	[%]	[Ω cm^2^]	[Ω cm^2^]	[%]	[%]
1:0	0.797	12.72	70.86	6.20	2153.2	7.99	7.36
0.9:0.1	0.795	15.38	70.85	5.17	1015.8	8.66	8.15
0.8:0.2	0.793	15.80	70.67	5.03	1057.2	8.85	8.34
0.7:0.3	0.784	16.06	72.08	4.55	908.3	9.08	8.70
0.6:0.4	0.778	16.57	68.02	5.35	933.2	8.77	8.15
0.5:0.5	0.769	16.39	62.77	5.98	553.8	7.91	7.67

## Data Availability

The original contributions presented in this study are included in the article/Supplementary Material. Further inquiries can be directed to the corresponding author.

## Supplementary Materials

The following supporting information can be downloaded at: https://www.mdpi.com/article/10.3390/mi17010097/s1, Figure S1: J-V characteristics of hole-only devices by using SCLC models; Figure S2: Morphological characteristics of ternary blend films; Figure S3: XRD analysis of DTS(FBTTh_2_)_2_:PBDTTT-EFT:PC_71_BM films; Figure S4: AFM height images of DTS(FBTTh_2_)_2_:PBDTTT-EFT:PC_71_BM films; Table S1: Thickness variation in DTS(FBTTh_2_)_2_:PBDTTT-EFT:PC_71_BM films.; Table S2: Hole mobility in each ternary condition which was calculated by the Mott–Gurney law.
