# Supplementary material for: Enhanced Photovoltaic Performance of Ternary Small Molecule/Polymer Bulk Heterojunction Solar Cells
Source: Micromachines (Basel). 2026 Jan 12;17(1):97. doi: 10.3390/mi17010097 (PMC12844427; doi:10.3390/mi17010097)
Supplement: Supplementary file 1 [file micromachines-17-00097-s001.zip › micromachines-4019045-supplementary.pdf]

micromachines

Enhanced Photovoltaic Performance of Ternary Small Molecule/Polymer Bulk Heterojunction Solar Cells

Soo Ah Nam [1], Jinwoo Lee [2] and Joonwon Lim [2,3,*]

1 Department of Materials Science and Engineering, Korea Advanced Institute of Science & Technology (KAIST), Daejeon 34141, Republic of Korea
2 Department of Information Display, College of Sciences, Kyung Hee University, Seoul 02447, Republic of Korea
3 KHU-KIST Department of Converging Science and Technology, Kyung Hee University, Seoul 02447, Republic of Korea
* Correspondence: joonwon.lim@khu.ac.kr

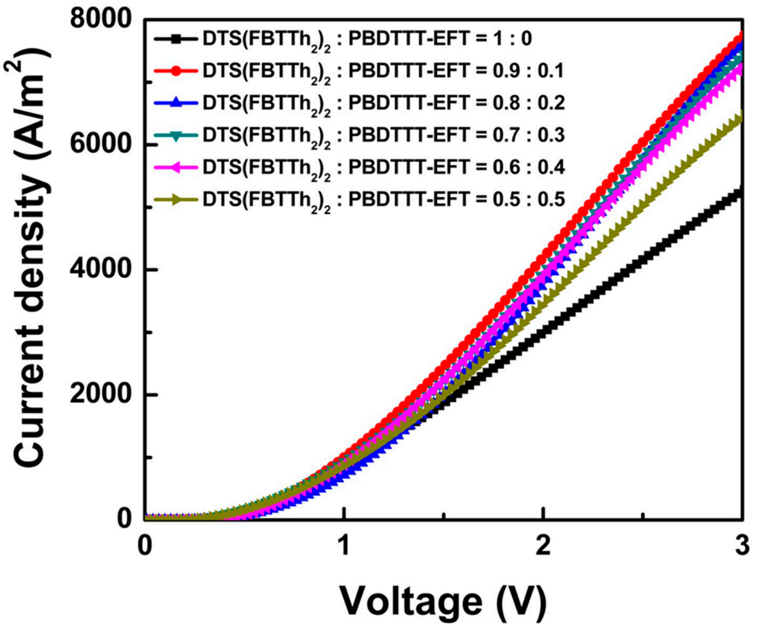

Figure S1. J-V characteristics of hole-only devices by using SCLC models.

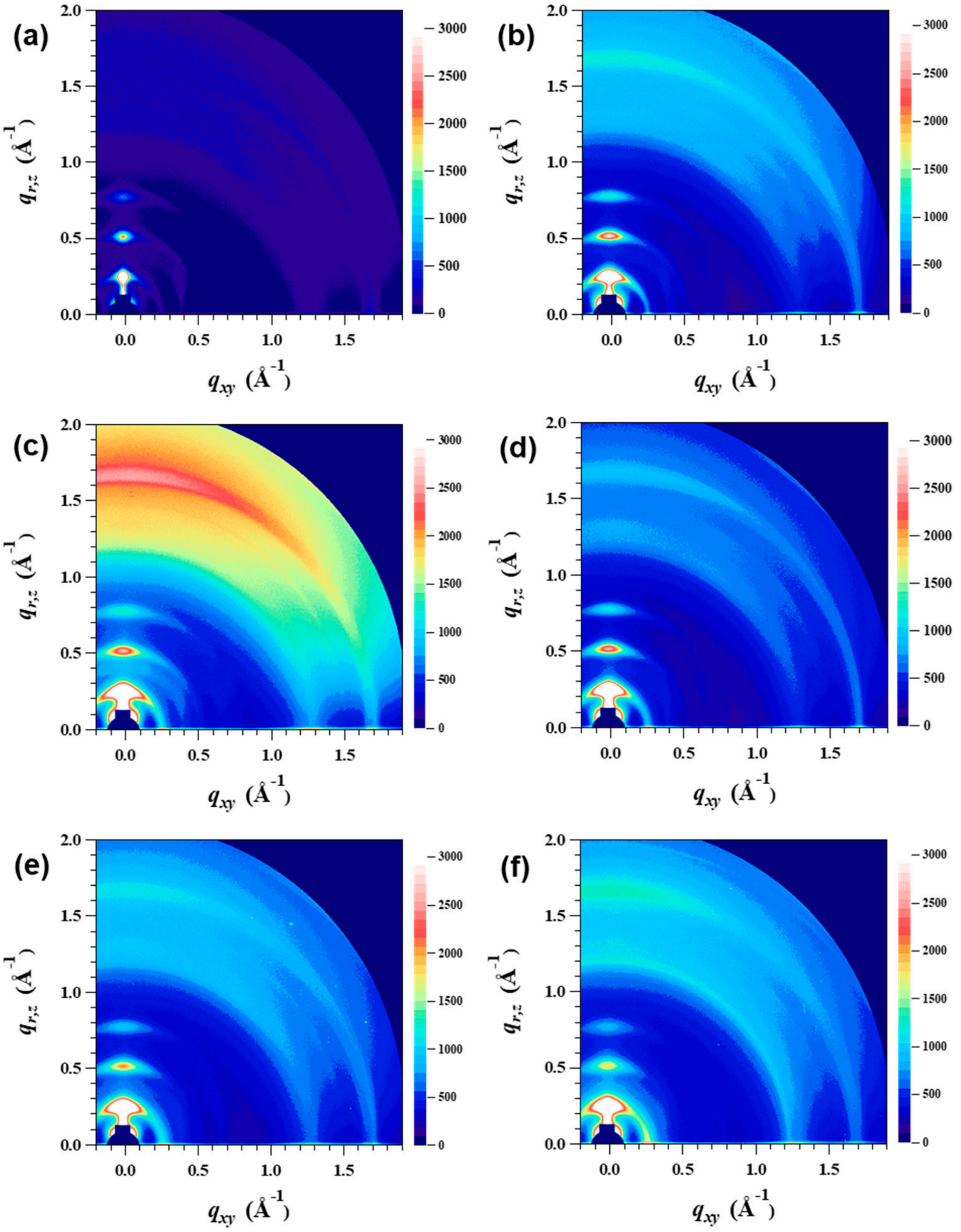

Figure S2. Morphological characteristics of ternary blend films. 2D GIWAXS analysis of ternary blend solar cell films. (a) DTS(FBTTh$_2$)$_2$:PBDTTT-EFT = 1:0, (b) DTS(FBTTh$_2$)$_2$:PBDTTT-EFT = 0.9:0.1 (c) DTS(FBTTh$_2$)$_2$:PBDTTT-EFT = 0.8:0.2 (d) DTS(FBTTh$_2$)$_2$:PBDTTT-EFT = 0.7:0.3 (e) DTS(FBTTh$_2$)$_2$:PBDTTT-EFT = 0.6:0.4 (f) DTS(FBTTh$_2$)$_2$:PBDTTT-EFT = 0.5:0.5.

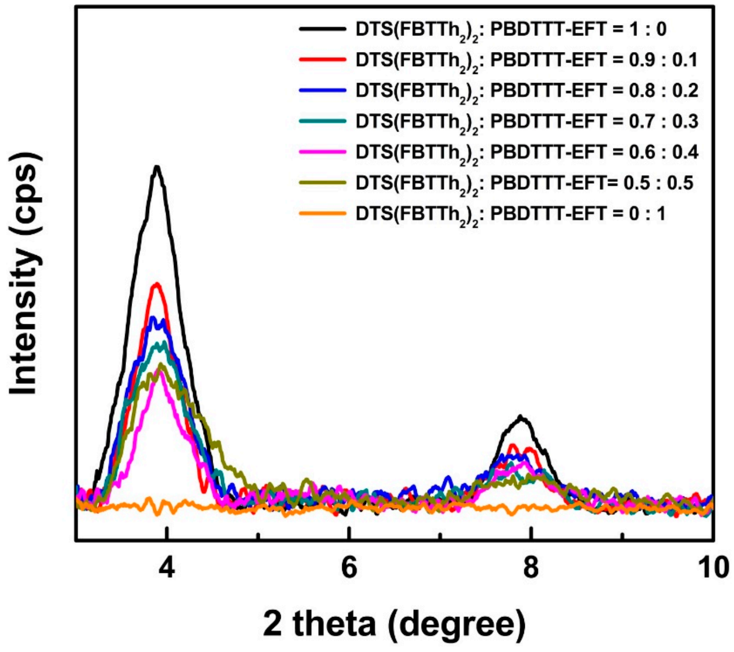

Figure S3. XRD analysis of DTS(FBTTh$_2$)$_2$:PBDTTT-EFT:PC$_{71}$BM films.

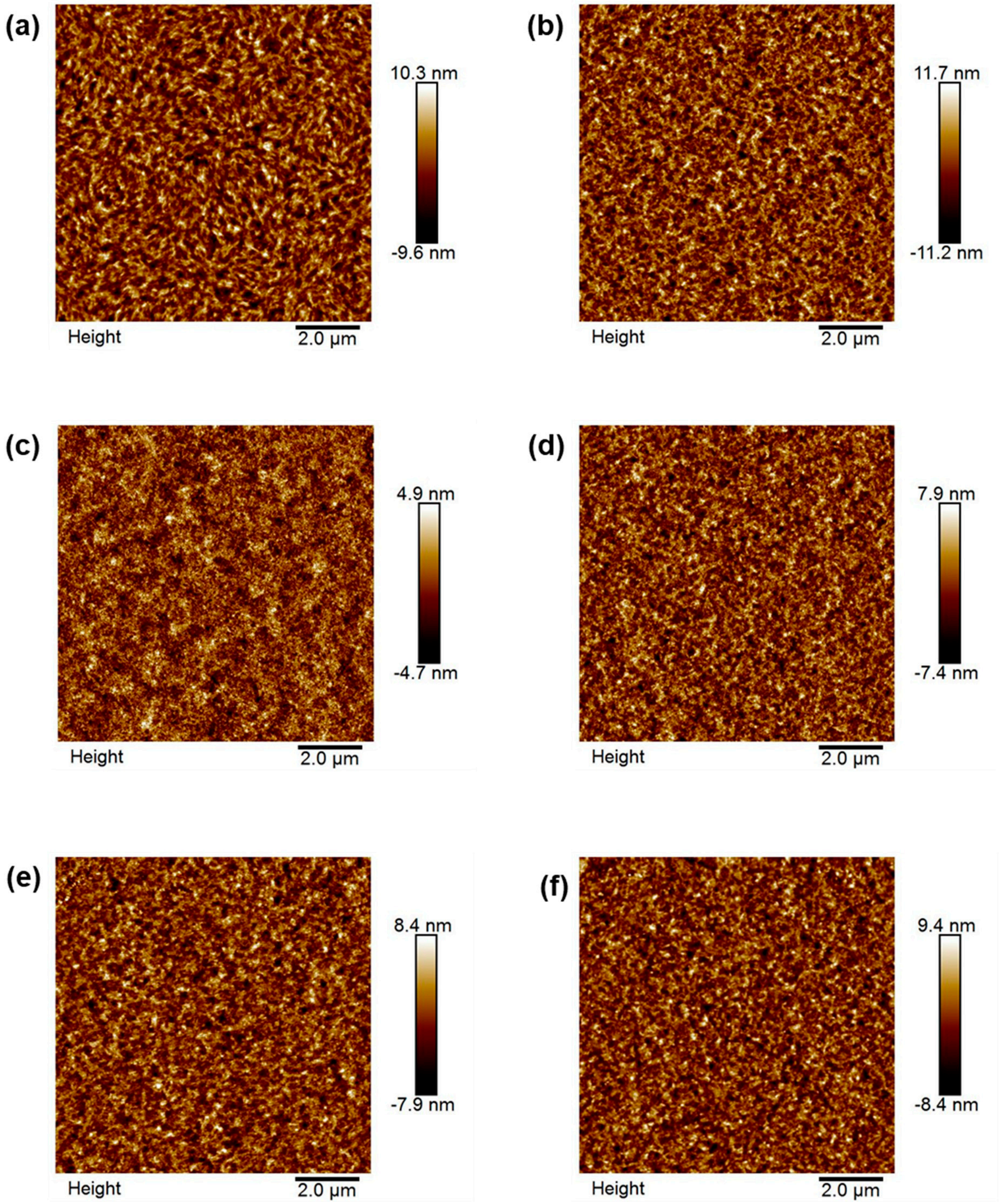

Figure S4. AFM height images of DTS(FBTTh$_2$)$_2$:PBDTTT-EFT:PC$_{71}$BM films. (a) DTS(FBTTh$_2$)$_2$:PBDTTT-EFT = 1:0, (b) DTS(FBTTh$_2$)$_2$:PBDTTT-EFT = 0.9:0.1 (c) DTS(FBTTh$_2$)$_2$:PBDTTT-EFT = 0.8:0.2 (d) DTS(FBTTh$_2$)$_2$:PBDTTT-EFT = 0.7:0.3 (e) DTS(FBTTh$_2$)$_2$:PBDTTT-EFT = 0.6:0.4 (f) DTS(FBTTh$_2$)$_2$:PBDTTT-EFT = 0.5:0.5

Table S1. Thickness variation of DTS(FBTTh$_2$)$_2$:PBDTTT-EFT:PC$_{71}$BM films.

DTS(FBTTh$_2$)$_2$:PBDTTT-EFT	Thickness [nm]
**1:0**	**109**
**0.9:0.1**	**119**
**0.8:0.2**	**133**
**0.7:0.3**	**146**
**0.6:0.4**	**162**
**0.5:0.5**	**175**

Table S2. Hole mobility in each ternary condition which was calculated by Mott-gurney law.

DTS(FBTTh$_2$)$_2$:PBDTTT-EFT	Hole mobility μ_h (cm^2 V^{-1} s^{-1})
**1:0**	3.90×10^{-4}
**0.9:0.1**	6.34×10^{-4}
**0.8:0.2**	7.69×10^{-4}
**0.7:0.3**	1.07×10^{-3}
**0.6:0.4**	1.46×10^{-3}
**0.5:0.5**	1.61×10^{-3}
